# Rapid differentiation of epithelial cell types in aged biological samples using autofluorescence and morphological signatures

**DOI:** 10.1371/journal.pone.0197701

**Published:** 2018-05-17

**Authors:** Emily R. Brocato, M. Katherine Philpott, Catherine C. Connon, Christopher J. Ehrhardt

**Affiliations:** Department of Forensic Science, Virginia Commonwealth University, Richmond, VA, United States of America; Institute of Human Virology, UNITED STATES

## Abstract

Establishing the tissue source of epithelial cells within a biological sample is an important capability for forensic laboratories. In this study we used Imaging Flow Cytometry (IFC) to analyze individual cells recovered from buccal, epidermal, and vaginal samples that had been dried between 24 hours and more than eight weeks. Measurements capturing the size, shape, and fluorescent properties of cells were collected in an automated manner and then used to build a multivariate statistical framework for differentiating cells based on tissue type. Results showed that epidermal cells could be distinguished from vaginal and buccal cells using a discriminant function analysis of IFC measurements with an average classification accuracy of ~94%. Ultimately, cellular measurements such as these, which can be obtained non-destructively, may provide probative information for many types of biological samples and complement results from standard genetic profiling techniques.

## Introduction

Characterizing the type of cells present in biological evidence and, therefore, the tissue they originated from within the body, can assist with crime reconstructions and downstream DNA profiling methods. Traditionally, caseworking methods for determining tissue source are based on microchemical and/or enzymatic reactions targeted toward proteins within bodily fluids, which have limited sensitivity and/or specificity. Recently, there has been considerable research into biomolecular markers for tissue identification. These include mRNA transcripts [1], micro-RNAs [2,3], proteomics [4], and DNA methylation patterns [5]. Although promising, the specificity of many of these systems is still being investigated and interpretation can require complex bioinformatic workflows.

In contrast, few forensic techniques have utilized morphological or intrinsic biochemical differences to differentiate between cells from different tissues, particularly epithelial cells. This is likely due to the laborious nature of microscopic characterizations or the need for tissue-specific antibody probes which have limited success on dried or compromised samples [6,7]. One potential strategy to address these challenges is the use of Imaging Flow Cytometry (IFC). In IFC, conventional flow cytometry analysis whereby the optical properties of individual cells are interrogated with lasers at set wavelengths, is combined with fluorescence and bright field imaging of those same cell events. IFC is routinely used in biomedical and clinical research for identification of unusual cell types as well as high resolution surveys of both cellular and sub-cellular processes [8]. The primary advantage of IFC over conventional microscopic analysis is that images of single cells are collected in a high throughput manner (as many as hundreds per second) and at multiple fluorescence channels simultaneously. The resulting multivariate data streams can therefore be used to compare profiles between individual cells or between larger populations. For forensic applications, another potential advantage of IFC is that it is an inherently non-destructive technique, with the possibility of collecting all cells after analysis for DNA profiling or other biological characterizations.

In this study we tested whether IFC could be used to differentiate epithelial cells from three separate tissue sources—buccal, touch epidermal, and vaginal—based on autofluorescence and morphological signatures. Identifying the presence of one or more of these cell types in a biological sample when combined with DNA profiling results may be useful when evaluating either single source or mixture samples in light of competing prosecution and defense propositions to explain the presence (or absence) of particular individuals’ DNA (e.g., claims of sexual assault versus denial of such activity and suggestions of indirect transfer). Additionally, because DNA yield has been observed to systematically vary between epidermal cells and other types of epithelial tissue [9], determining the presence and relative quantities of each cell type can help direct downstream DNA profiling efforts. We conducted an initial IFC survey of each epithelial cell type by analyzing existing biological specimens from a forensic sample repository consisting of ten donors per cell type. To assess the robustness and consistency of IFC signatures against samples approximating those that would be encountered during forensic casework (e.g., samples collected different lengths of time after deposition, and/or stored for different lengths of time prior to analysis), the samples were “aged” for different amounts of time prior to analysis, ranging from ~24 hours to more than eight weeks.

## Methods

### Sample collection and preparation

Buccal and epidermal samples were obtained from male and female volunteers pursuant to the Virginia Commonwealth University Institutional Review Board (VCU-IRB) approved protocol ID#HM20000454_CR3. Written informed consent was obtained from all participants for this study. For buccal samples, ten volunteers were asked to swab the inside of cheek for 30 seconds. Swabs were left to dry for between 24 hours and 6 days. Dried and fresh swabs were processed in the same manner. For epidermal samples, ten individuals (six of whom were buccal cell donors) were asked to hold/rub a conical tube (P/N 229421; Celltreat Scientific; Pepperell, MA) for five minutes to deposit cells. Tubes were then left out for 24 hours to 5 days to dry before collecting cells. Cells were collected from the surface with one sterile, pre-wetted swab, and one sterile, dry swab.

Vaginal cell samples were obtained from an existing sample repository at Virginia Commonwealth University. Samples were collected pursuant to VCU-IRB approved protocol ID#HM20002931_Ame2. Volunteers were asked to swab the inside of the vaginal cavity, and swabs were dried and stored at room temperature until analysis. Storage times ranged from 72 hours to approximately eight weeks.

All collection swabs were eluted in 1 mL of 1x Cell Staining Buffer (P/N 420201; Biolegend; San Diego, CA), and gently vortexed for 10 seconds. Samples were centrifuged at 1500 × g at 4°C for 5 minutes. The supernatant was discarded, and the cell pellets were dissolved in 100 uL of 1x Cell Staining Buffer for imaging flow cytometry. A list of all donor samples used in this study and their respective drying times are provided in S1 Table. The IRB approved protocols required the donors to confirm that they were over 18 years of age, but did not require that their age be recorded.

### Imaging Flow Cytometry and statistical analysis

All samples were analyzed using an Amnis® Imagestream X Mark II (EMD Millipore; Burlington, MA) equipped with 405nm, 488nm, 561nm, and 642nm lasers. Laser voltages for all tests were set at 120mW, 100mW, 100mW and 150mW, respectively. Images of individual events were captured in five detector channels labeled: 1 (430-505nm), 2 (505-560nm), 3 (560-595nm), 5 (640-745nm), and 6 (745-780nm). Channel 4 was used to capture Brightfield images. Magnification was set at 40x and autofocus was enabled so that the focus varied with cell size. Examples of cell images collected across multiple wavelengths are provided in S1 Fig. Scatterplots of Aspect Ratio and Area values for a sample of each cell type are also given in S1 Fig (aspect ratio and area are comparable to forward scatter/side scatter measurements collected with conventional flow cytometry instrumentation). Raw image files (.rif) were then imported into IDEAS® Software (EMD Millipore; Burlington, MA). Display Width and Display Height were changed to 120x120 pixels for each image. The ‘Shape Change Wizard’ option in Ideas was used to select focused cells on a Gradient RMS_M04Ch04 x Normalized Frequency histogram. Once the data was filtered for focused cells, single cells were selected on an Area_M04 x Aspect Ratio_M04 scatterplot. This was to ensure that cell aggregates were not incorporated into the downstream analysis.

Data for individual cell events were collected for 17 different features: area, aspect ratio, aspect ratio intensity, contrast, intensity, mean pixel, median pixel, max pixel, length, width, height, brightness detail intensity (‘R3’ pixel increment), raw centroid X, raw centroid Y, and circularity. These feature measurements were collected across multiple detector channels (i.e., fluorescence and brightfield wavelengths) with the exception of measurements that could only be determined from brightfield images such as centroid X/Y and circularity. This yielded a total of 88 measurements/variables collected for each cell. Cell yield varied across each of the study samples but did not appear to be correlated with tissue type, drying time, or individual donor. Most cell populations yielded between 200 and 400 cell images with nine samples providing between 80 and 200 images.

IFC measurement values were then imported into SPSS v23 (IBM, Inc. Chicago, IL). Differences in mean values between the three cell types were tested using a one-way ANOVA analysis with a Tukey HSD post-hoc test performed in SPSS. Next, multivariate differences among the three cell type groups were analyzed using a Discriminant Function Analysis (DFA) based on the within-group covariance matrix. We initially compared results from direct analysis of IFC measurements and those obtained from transforming the data first into principal components (PCs) and then conducting DFA on the PC scores. We found that the latter approach led to less differentiation in the canonical variate plot and poorer classification accuracy and thus used direct analysis of raw measurements. Different combinations of variables were initially tested based on their impact on group separation in the canonical variate plot and classification accuracy. Inclusion of all variables in the analysis resulted in the greatest degree of separation in the canonical variate plot and the highest rate of accurate classifications.

Source IFC data (.rif files) and extracted cellular measurements used for statistical analyses are available through a Figshare repository; doi: 10.6084/m9.figshare.5847933.

## Results

**Fig 1:**
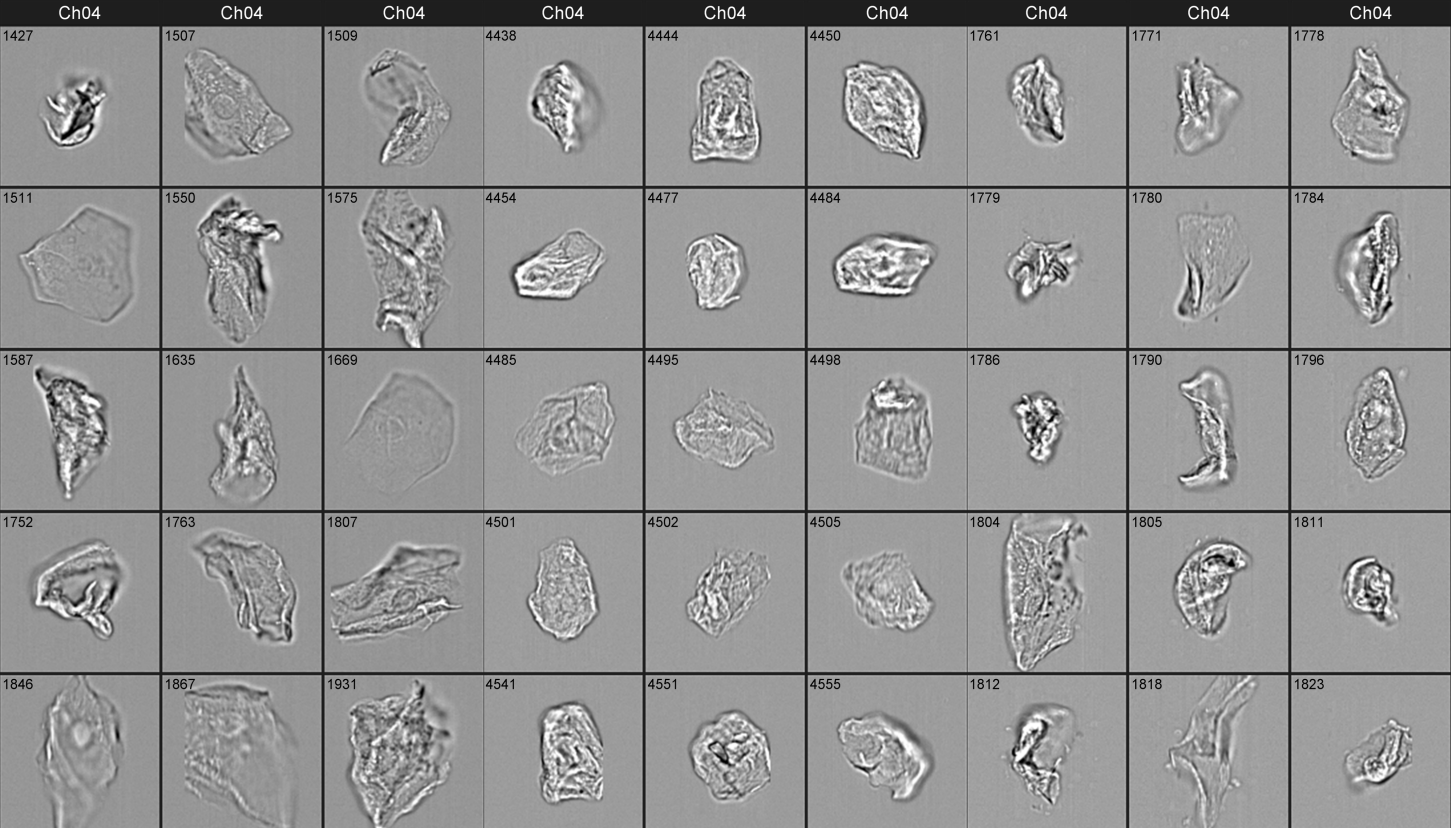
Image gallery for three epithelial cell tissue sources. IFC Brightfield images for buccal cells (columns 1–3), epidermal cells (columns 4–6), and vaginal cells (columns 7–9). Each image frame is 50 μm x 50 μm. Object identifiers are included with each image.

We first tested whether IFC could be used to distinguish cells from the three different epithelial tissue sources. During image collection and processing, we noted some general qualitative differences between images from each of the three cell types. For example, circular features with a size consistent with nuclei (~8μm), were observed in the center of many of the buccal cells and vaginal cells (e.g., Images 1507, 1796, respectively, Fig 1), while they were rarely observed in epidermal cell images. Buccal and vaginal cells were generally larger in size, >40 μm compared to epidermal cells, which were ~20–50 μm although we noted some size overlap between cell sources. This could be due in part from the folding or degradation of buccal and vaginal cells during drying or sampling prior to IFC. Epidermal cells generally exhibited higher contrast features in brightfield images compared to buccal or vaginal cells.

For the 264 pairwise comparisons between group means (88 variables and three sample groups), only 42 yielded p-values greater than 0.01, with the vast majority showing p values less than 0.0001 (S2 Table). Of note were differences in means for circularity (7.8 epidermal, 4.1 buccal, 4.3 vaginal), intensity (e.g., in 430-505nm channel 3x10^5^ RFU epidermal, 6x10^4^ RFU buccal, 5x10^4^ RFU vaginal), and brightness detail (e.g., in 403–505 nm channel 1x10^4^ RFU epidermal, 9x10^3^ buccal, 7x10^3^ RFU vaginal). However, the range of values for each cell group showed a high degree of overlap across the three cell types (Boxplots in S2 Fig). Similarly, most variables showed large standard deviations for each cell type, with coefficients of variation for individual measurements ranging from ~20% to more than 280%.

**Fig 2:**
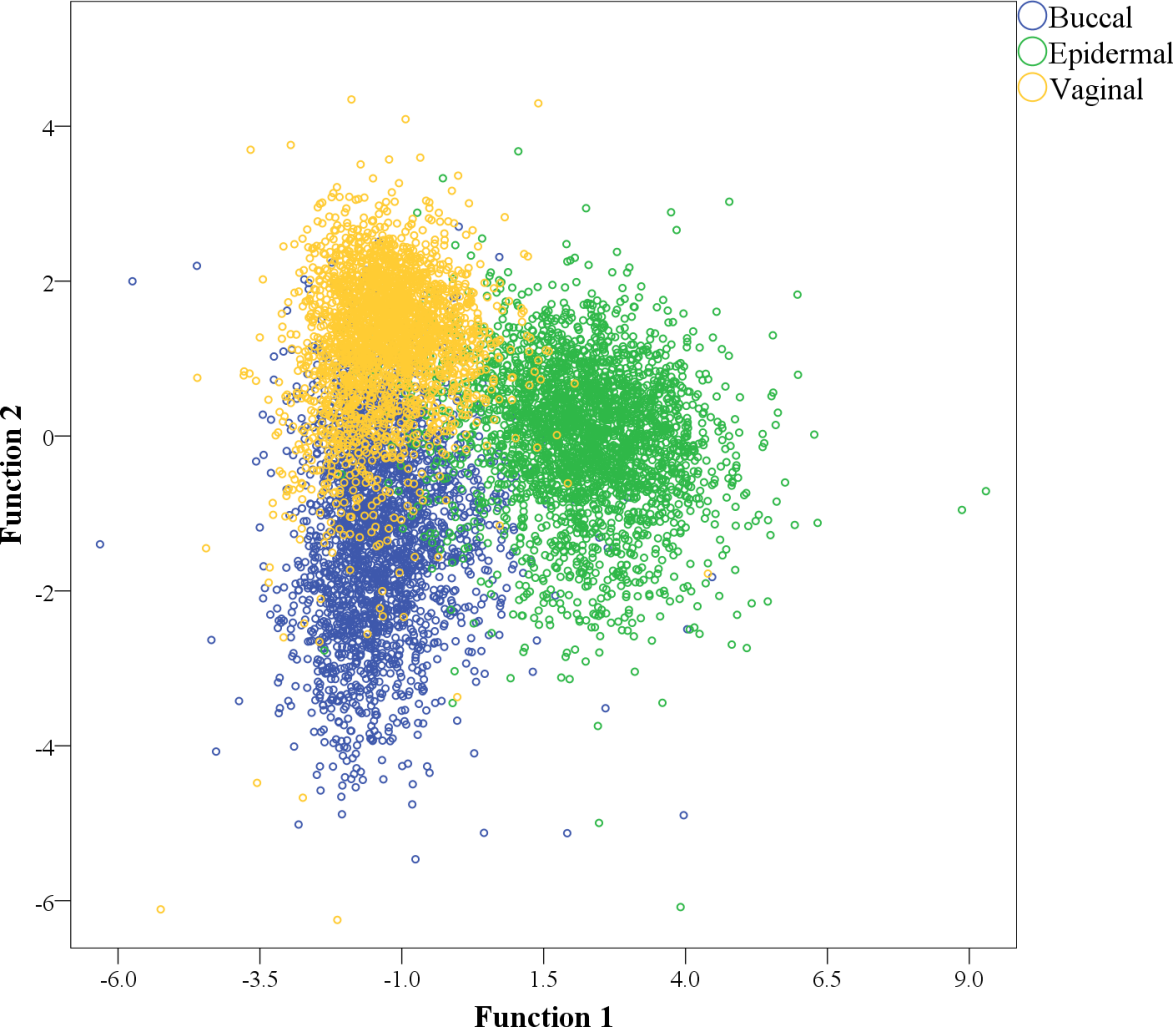
Discriminant Function Analysis of epithelial cells from three tissue sources using IFC variables. The first discriminant function (x-axis) accounted for ~74% of the between group variation and the second discriminant function (y-axis) accounted for ~26%.

In order to determine whether the observed variation in IFC measurements could be used to differentiate cell types, we employed Discriminant Function Analysis (DFA) as a supervised multivariate technique to model variation between groups. In DFA, linear combinations of the original variables are constructed (i.e., canonical variates) such that the variation between user-defined sample groups is maximized and within group variation is minimized. DFA is a well-established technique with demonstrated applications for other forensic signature systems [10–12]. For this dataset, the primary advantages of DFA are that differences in measurement scales across variables do not impact the analysis and it is relatively robust to non-normally distributed data [13]. Additionally, the canonical variates generated with DFA can be used to classify individual samples into one of the user-defined groups. For this study we used DFA to initially examine multivariate differences between groups. A DFA plot of all IFC measurements from the three cell types showed distinct separation between buccal, epidermal, and vaginal cell populations (Fig 2). Multivariate differences between groups were statistically significant, Wilk’s Lambda = 0.114, p<0.001. Some overlap is observed among the sample groups on the DFA plot, in particular between buccal and vaginal cell groups. A leave-one-out (LOO) classification on individual cell images for each of the three groups and all 30 cell populations showed an overall classification accuracy of ~90%.

Next we used DFA-based algorithms to classify entire donor cell populations into one of the three cell groups in a blinded fashion to determine the accuracy and robustness of this approach for identifying cell types from an unknown forensic sample. This was accomplished by withholding a given donor cell population from the DFA and classifying each cell image into one of the three epithelial cell types based on information from the remaining contributor cell populations. Classification results for cell populations against three tissue types are given in Table 1. In general, epidermal cells showed the highest overall classification accuracy (88%) with six of the ten donor cell populations having accuracies over 90%. Only one cell population, P22, was below 80%. Buccal and vaginal cell populations yielded lower overall classification rates, 72% and 75% respectively (Table 1). Interestingly, classification accuracies were highly variable across individual cell populations for these two groups, with buccal cells ranging between 24% and 96% and vaginal cells ranging between 26% and 95%.

**Table 1:** Classification test of buccal, epidermal and vaginal cells.

			Predicted Cell Type		
Cell Type	Contributor ID	Buccal	Epidermal	Vaginal	Classification %
Buccal	L49	254	23	30	83
Buccal	I66	244	2	69	78
Buccal	C58	198	13	49	76
Buccal	R47	157	6	1	96
Buccal	5001	214	3	134	61
Buccal	B21	185	1	15	92
Buccal	N08	130	4	6	93
Buccal	Y60	126	12	59	64
Buccal	Z32	138	16	48	68
Buccal	5034	59	3	183	24
**Total**	**—**	**1705**	**83**	**594**	**72**
Epidermal	Q17	3	190	10	94
Epidermal	Z32	18	175	10	86
Epidermal	Y60	3	189	10	94
Epidermal	K36	0	421	12	97
Epidermal	P22	24	216	123	60
Epidermal	S95	2	76	6	90
Epidermal	I66	15	295	26	88
Epidermal	L49	3	347	1	99
Epidermal	R47	55	247	0	82
Epidermal	N08	7	286	10	94
**Total**	**—**	**130**	**2442**	**208**	**88**
Vaginal	2368	171	7	200	53
Vaginal	4017	7	9	296	95
Vaginal	1022	37	8	312	87
Vaginal	1028	96	1	35	26
Vaginal	1031	1	22	344	94
Vaginal	5020	49	1	118	70
Vaginal	5021	4	41	92	67
Vaginal	5005	12	72	79	47
Vaginal	4502	20	0	180	90
Vaginal	4504	80	0	222	74
**Total**	**—**	**477**	**161**	**1878**	**75**

In an attempt to improve the classification accuracy for each cell type, individual cell populations were also tested with two-group classification schemes where one tissue group was excluded completely from the analysis, i.e., buccal cells against epidermal cells; vaginal cells against epidermal cells; and buccal cells against vaginal cells (Tables 2–4 respectively). Simplified classification schemes could be run subsequent to the original classification to help identify samples assigned to one of the closely related sample groups, i.e., a cell image classified as a buccal cell in the three group DFA could then be run against a two group DFA containing only buccal and vaginal cells. Tiered or successive DFA analyses have been described for other types of forensic samples [10,14]. Additionally, two group comparisons could approximate caseworking scenarios in which one of the epithelial cell types could be ruled out *a fortiori* for an unknown cell population. Results from two-group DFA generally showed improved classification accuracy. Buccal and epidermal cell populations could be differentiated with the highest accuracy (~94%). The lowest classification rate of individual donor cell populations in this comparison was 80% (P22, Epidermal) with the majority of cell populations exhibiting classification accuracy of 95% or higher (Table 2). The vaginal-epidermal cell classifications showed comparable results with an overall classification accuracy of ~91%. Two individual cell populations in this scheme exhibited markedly lower success rates (P22 epidermal 63% and 5005 vaginal 32%). However, the remaining cell populations had classification accuracy >80% with the majority >95% (Table 3). Less differentiation was observed between buccal and vaginal cells with an overall classification accuracy of 78% (Table 4). Seven donor cell populations still showed accuracies greater than 95% and three donor cell populations were below 60% accuracy (e.g., 5034, Buccal; 2368 Vaginal; 1028 Vaginal).

**Table 2:** Classification test of buccal vs. touch epidermal cells.

		Predicted Cell Type		
Cell Type	Contributor ID	Buccal	Epidermal	Classification %
Buccal	L49	287	20	94
Buccal	I66	308	7	98
Buccal	C58	229	31	88
Buccal	R47	151	13	92
Buccal	5001	348	3	99
Buccal	B21	198	3	98
Buccal	N08	135	5	96
Buccal	Y60	174	22	89
Buccal	Z32	174	27	87
Buccal	5034	233	11	96
**Total**	**—**	**2237**	**142**	**94**
Epidermal	N08	3	300	99
Epidermal	R47	32	270	89
Epidermal	L49	2	349	99
Epidermal	I66	10	326	97
Epidermal	S95	3	81	96
Epidermal	P22	71	292	80
Epidermal	K36	1	432	100
Epidermal	Y60	4	198	98
Epidermal	Z32	19	184	91
Epidermal	Q17	3	200	98
**Total**	**—**	**148**	**2632**	**95**

**Table 3:** Classification test of vaginal vs. touch epidermal cells.

		Predicted Cell Type		
Cell Type	Contributor ID	Vaginal	Epidermal	Classification %
Vaginal	2368	329	49	87
Vaginal	4017	301	11	96
Vaginal	1022	336	21	94
Vaginal	1028	113	19	86
Vaginal	1031	351	16	96
Vaginal	5020	163	5	97
Vaginal	5021	119	18	87
Vaginal	5005	54	114	32
Vaginal	4502	200	0	100
Vaginal	4504	302	0	100
**Total**	**—**	**2268**	**253**	**90**
Epidermal	Q17	10	193	95
Epidermal	Z32	12	191	94
Epidermal	Y60	4	198	98
Epidermal	K36	15	418	96
Epidermal	P22	134	229	63
Epidermal	S95	6	78	93
Epidermal	I66	20	316	94
Epidermal	L49	2	349	99
Epidermal	R47	2	300	99
Epidermal	N08	5	298	98
**Total**	**—**	**210**	**2570**	**92**

**Table 4:** Classification test of buccal vs. vaginal cells.

		Predicted Cell Type		
Cell Type	Contributor ID	Buccal	Vaginal	Classification %
Buccal	L49	294	13	96
Buccal	I66	267	48	85
Buccal	C58	227	33	87
Buccal	R47	164	0	100
Buccal	5001	188	163	54
Buccal	B21	195	6	97
Buccal	N08	133	7	95
Buccal	Y60	157	39	80
Buccal	Z32	146	55	73
Buccal	5034	31	213	13
**Total**	**—**	**1802**	**577**	**76**
Vaginal	2368	250	128	34
Vaginal	4017	19	293	94
Vaginal	1022	8	349	98
Vaginal	1028	56	76	58
Vaginal	1031	3	364	99
Vaginal	5020	46	122	73
Vaginal	5021	0	137	100
Vaginal	5005	17	151	90
Vaginal	4502	16	184	92
Vaginal	4504	66	236	78
**Total**	**—**	**481**	**2040**	**81**

To investigate whether the DFA classification scheme can accurately assess the proportion of cell types in a two-person mixture, we created simulated mixtures by randomly sampling two donors’ cell images. These images were then classified into cell types using with the remaining contributor cell populations as the reference dataset for DFA. A 1:1 simulated mixture consisting of L49 (epidermal) and B21 (buccal) cell images was classified as 50% epidermal cells and 46% buccal cells, with the remaining 4% of images classifying as vaginal cells. Using the two-group classification scheme, the cell population was determined to be 50% epidermal cells and 50% buccal cells. Similar results were obtained for a 1:1 simulated mixture consisting of Q17 (epidermal) and 1031 (vaginal) cell images, with the population characterized as 49% epidermal cells, 49% vaginal cells and 2% buccal cells. The two group classification scheme estimated a cell population of 50% epidermal cells and 50% vaginal cells. Mixtures containing contributor populations that demonstrated lower classification accuracy in earlier experiments had lower success rates. For example, a 1:1 simulated mixture consisting of C58 (buccal) and R47 (epidermal) cell images classified as 42% epidermal cells, 51% buccal cells, and 7% vaginal cells.

## Discussion

Overall, the relatively high classification accuracy of epidermal cells against buccal cells and epidermal cells against vaginal cells (>90%) suggests that systematic differences in morphological and/or optical properties measured by IFC can be used to distinguish between epithelial cell types in these two comparison schemes. Further, measurement values can potentially be used to construct an analysis framework for characterizing unknown cell populations into one of these three sample groups. The observed variation between sloughed epidermal cells and buccal/vaginal cells is consistent with the intrinsic biochemical, structural, and morphological differences for cells originating from each tissue source. For example, shed epidermal cells are derived from the stratum corneum and characterized by a high degree of keratinization with few if any organelles and little intracellular DNA owing to the apoptotic processes occurring as cells migrate from the basal to the upper layers of the epidermis [15]. In contrast, buccal and vaginal cells are derived from less stratified epithelial tissue and may be only partially keratinized or unkeratinized. Although no studies to date have explicitly surveyed cellular differences between these three tissue sources using fluorescence signatures, previous work has shown that changes in cellular autofluorescence can be used to differentiate layers of epidermal tissue with different intracellular components (e.g., keratin, tryptophan, FAD) [16,17]. Additionally, the morphological and size differences detected with IFC (e.g., area and circularity measurements) are consistent with histological context of each cell type, i.e., shed epidermal cells hexagonal and ~20–50 μm, while buccal and vaginal cells are typically >40 μm with elongated shapes [18,19].

The overlap between cell sources shown in Fig 2 and misclassifications of individual cell images may be impacted by a number of factors. First, some similarities in fluorescence and/or morphological attributes are expected, particularly for buccal and vaginal cells given that both are derived from non-keratinized epithelial tissue. This is consistent with poorer classification accuracy of buccal-vaginal cell comparisons relative to buccal-epidermal and vaginal-epidermal (Table 4 vs. Tables 2 and 3 respectively). Second, cell populations in this data set represent a wide range of drying/exposure times prior to sampling and analysis. Levels of intrinsic fluorescence are likely to change with time owing to the degradation of cellular components such that specimens with longer periods of environmental exposure may be harder to distinguish from each other. Although there were no clear relationships between exposure time and misclassification rate (Tables 1–4) or position on the DFA plot (Fig 2), this should be systematically tested with future studies. A preliminary analysis of buccal cell populations from two donors, each aged for 3, 24, 48, and 72 hours, suggests that fluorescence and/or morphological features may change in a characteristic way over time (S3 Fig). Further study may permit the development of an analysis framework that allows for estimations of “age” of cell samples.

**Fig 3:**
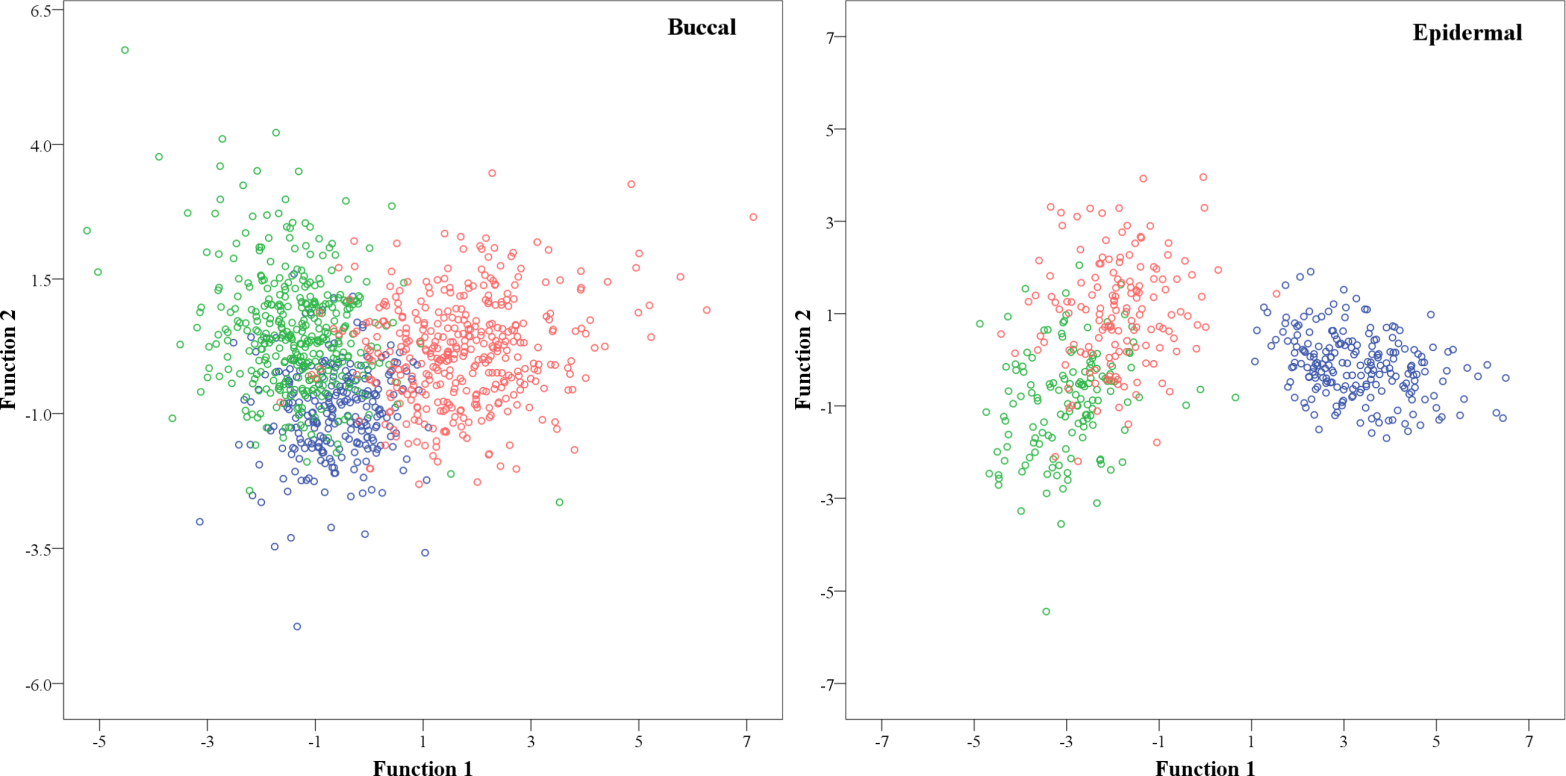
Discriminant function analysis of cell populations derived from different contributors for buccal and epidermal tissue sources. Buccal cells (left) were dried for 48 hours at room temperature and epidermal cell samples (right) were dried for 24 hours at room temperature prior to analysis. Red, green, blue circles represent three different individuals in each graph.

Another factor that could be contributing to misclassifications is inter-individual variation. Previous work from our group has shown that autofluorescence signatures in shed epidermal cells can vary between contributors, likely owing to the presence of exogenous materials associated with the cell [20]. Cell populations from different contributors of the same tissue type (epidermal or buccal) and drying time (24 or 48 hours, respectively) showed some separation in a preliminary DFA (Fig 3). It is also possible that the number of donor cell populations in this study did not adequately capture the full range of morphological and/or fluorescence variation that exists between contributors. Increasing the number of unique donor cell populations in the reference/comparison dataset will likely help to isolate any tissue-specific signatures that are present. Nevertheless, contributor-specific variation in IFC measurements is a potentially promising avenue of future research for this technique, particularly how it might be used for estimating the number of individual cell populations in a biological sample and/or facilitating front-end cell separation in a DNA profiling workflow.

It should also be noted that earlier studies have suggested that sex-specific differences in the size and morphology of epidermal cells may exist [18]. Although there were no obvious differences in classification accuracy or position on the DFA plot across male and female donors, IFC could be a viable approach for systematically testing for sex specific signatures in a larger dataset of epidermal cell populations.

Our goal with this study was to conduct an initial assessment of high-throughput analysis of autofluorescence and morphological signatures and its potential applications for characterizing epithelial cell types in an unknown biological sample. An important aspect of this workflow is that intrinsic properties of the cell are being analyzed and no biochemical or immunological stains or probes are required. High-throughput, single cell measurements combined with a multivariate classification framework were used to distinguish epidermal cells from other epithelial cell sources across a range of drying times with an overall high degree of accuracy. Although a range of factors may contribute to morphological or optical properties in any given sample (e.g., individual-specific signatures and degradation time), these results suggest that multivariate approaches may be used to extract tissue-specific signatures from biological samples. Future work should test alternative classification methods such as machine learning algorithms as well as different combinations of cellular measurements to maximize classification accuracy particularly for cell types with similar biochemical and physical properties but different source tissue, and eventually establish cell count thresholds for inferring the presence and/or proportions of one or more cell types in a biological sample. For mixture samples in particular, the ability to quantify epithelial cell types in a non-destructive and high-throughput manner at the front end of a DNA profiling workflow could improve methodological decision making during DNA analysis and interpretation, and ultimate results. We also note that the fluorescence and morphological signatures identified here could be detected and analyzed using other microscopy setups and open source software platforms (e.g., [21]) which may facilitate its use in caseworking laboratories.

## Supporting information

S1 Fig Imaging Flow Cytometry output. Top-Example data output from imaging flow cytometry analysis of buccal cell population. Each column corresponds to a different detector channel. Bottom-example scatterplots of area and aspect ratio for each cell type determined with IFC.
(TIF)

S2 Fig Box plots of feature values measured for individual cells within each tissue group. Group 1 = Buccal cells, Group 2 = Epidermal cells, Group 3 = Vaginal cells.
(PDF)

S3 Fig Discriminant function analysis of buccal cell populations from the same donor aged for different amounts of time. Left and right panels correspond to two separate individuals (I66 and L49 respectively).
(TIF)

S1 Table Tissue type and drying time for each sample. (DOCX)

S2 Table ANOVA (Tukey HSD) for IFC measurements across cell types. (PDF)
