# Supplementary figures and images for: Rapid differentiation of epithelial cell types in aged biological samples using autofluorescence and morphological signatures
Source: PLoS One. 2018 May 17;13(5):e0197701. doi: 10.1371/journal.pone.0197701 (PMC5957390; doi:10.1371/journal.pone.0197701)

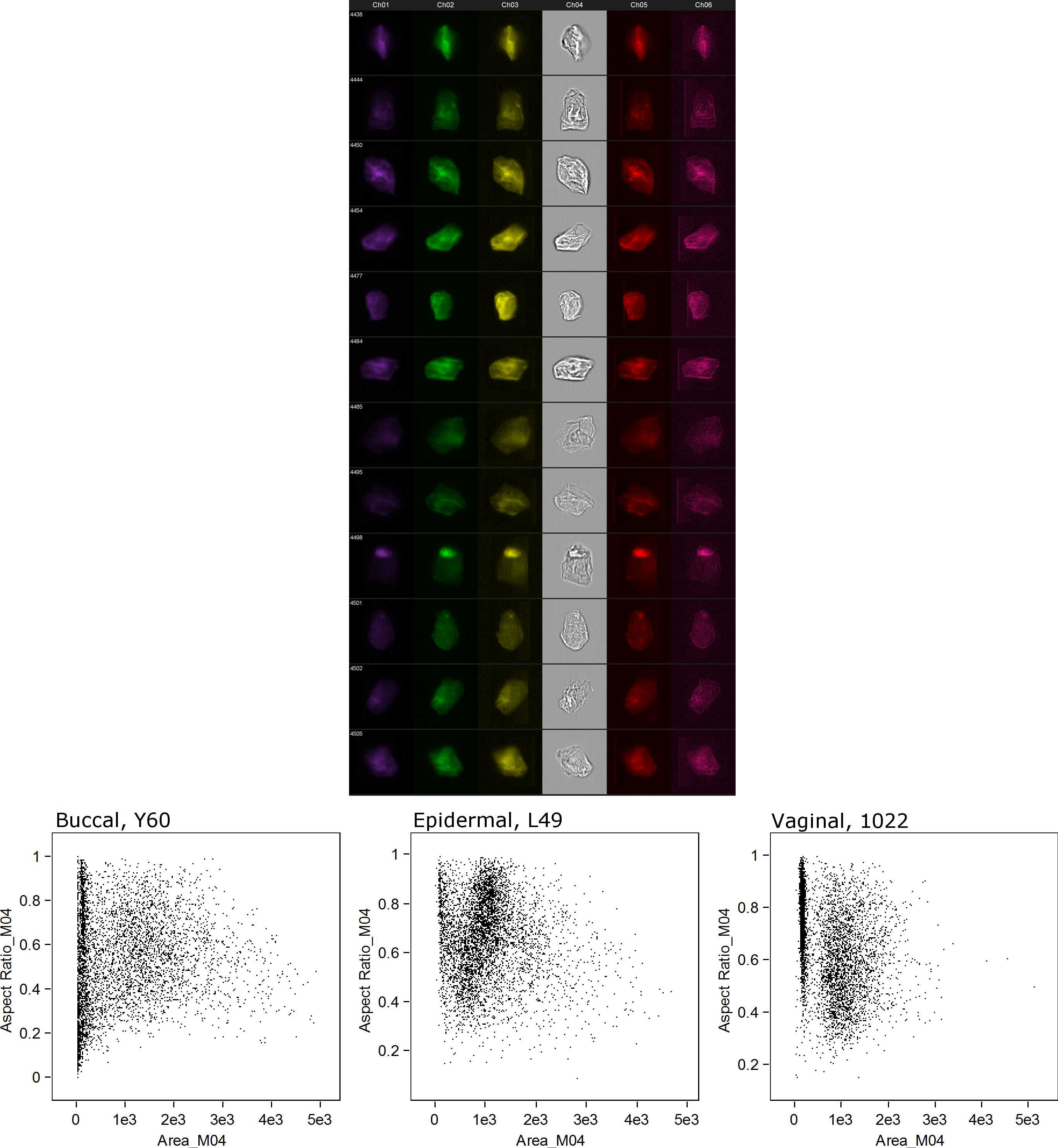

Supplement: S1 Fig — Top-Example data output from imaging flow cytometry analysis of buccal cell population. Each column corresponds to a different detector channel. Bottom-example scatterplots of area and aspect ratio for each cell type determined with IFC. (TIF) [file pone.0197701.s001.tif]

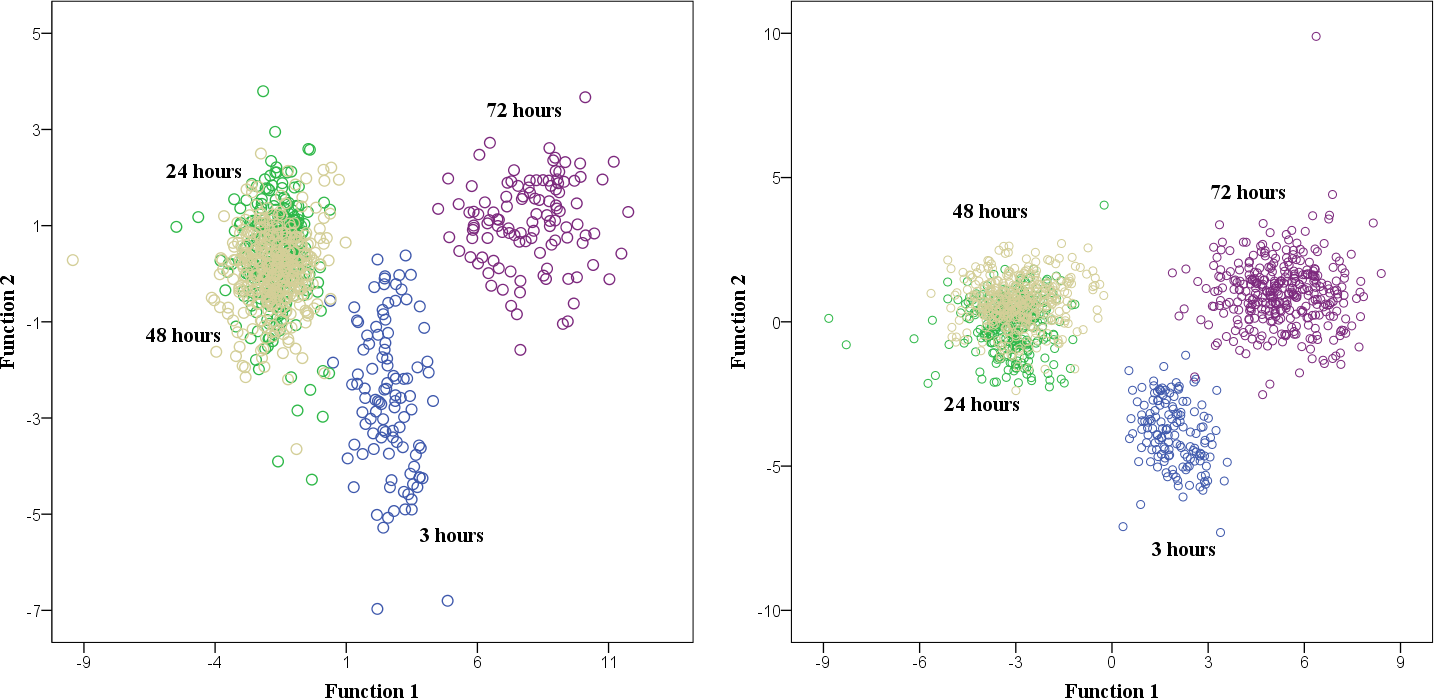

Supplement: S3 Fig — Left and right panels correspond to two separate individuals (I66 and L49 respectively). (TIF) [file pone.0197701.s003.tif]
