# Supplementary material for: Rapid differentiation of epithelial cell types in aged biological samples using autofluorescence and morphological signatures
Source: PLoS One. 2018 May 17;13(5):e0197701. doi: 10.1371/journal.pone.0197701 (PMC5957390; doi:10.1371/journal.pone.0197701)

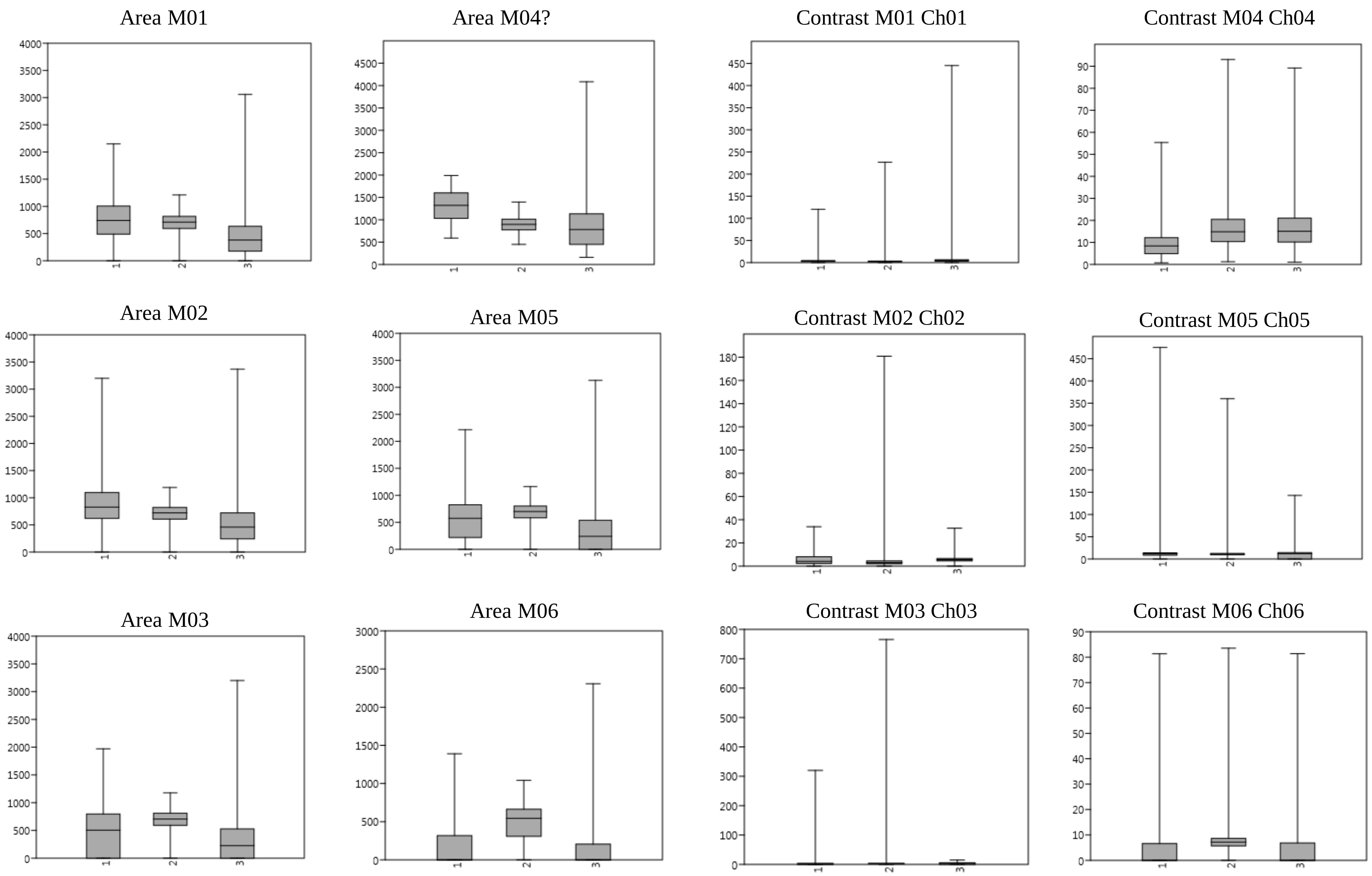
Area M01
Area M04?
Contrast M01 Ch01
Contrast M04 Ch04
Area M02
Area M05
Contrast M02 Ch02
Contrast M05 Ch05
Area M03
Area M06
Contrast M03 Ch03
Contrast M06 Ch06

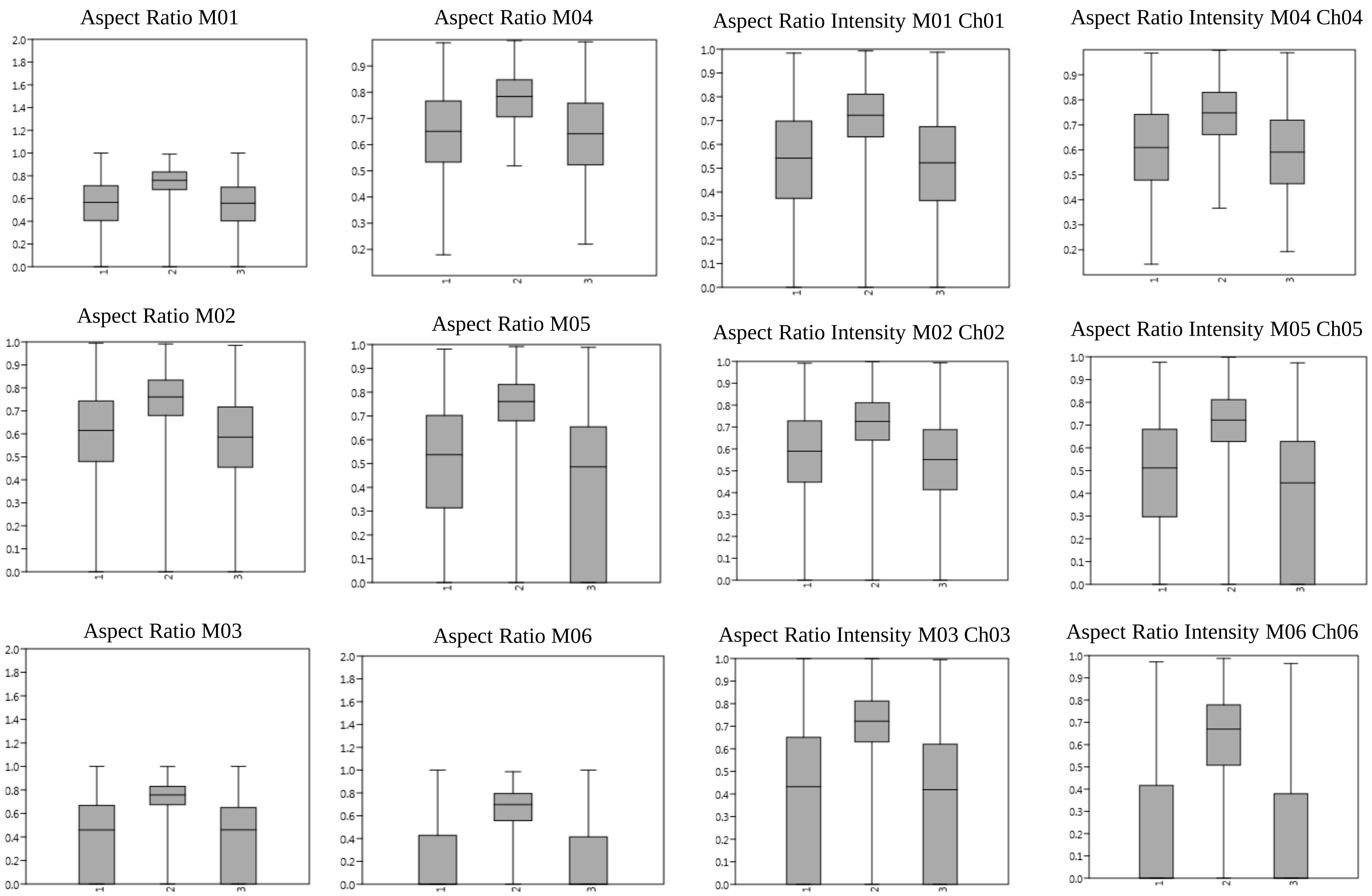
Aspect Ratio M01
Aspect Ratio M04
Aspect Ratio Intensity M01 Ch01
Aspect Ratio Intensity M04 Ch04
Aspect Ratio M02
Aspect Ratio M05
Aspect Ratio Intensity M02 Ch02
Aspect Ratio Intensity M05 Ch05
Aspect Ratio M03
Aspect Ratio M06
Aspect Ratio Intensity M03 Ch03
Aspect Ratio Intensity M06 Ch06

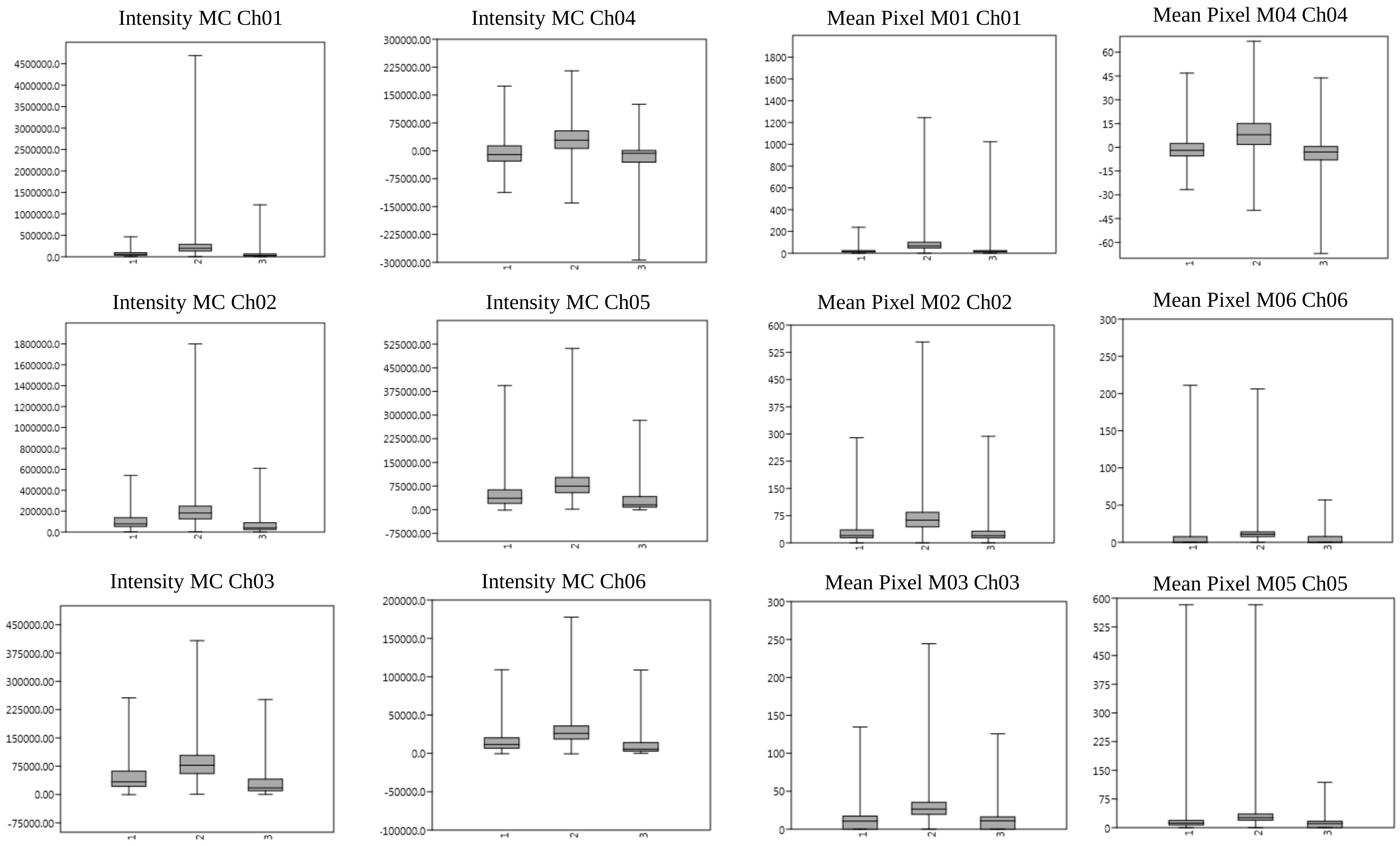

Intensity MC Ch01
4500000.0
4000000.0
3500000.0
3000000.0
2500000.0
2000000.0
1500000.0
1000000.0
500000.0
0.0
1
2
3
Intensity MC Ch04
300000.00
225000.00
150000.00
75000.00
0.00
-75000.00
-150000.00
-225000.00
-300000.00
1
2
3
Mean Pixel M01 Ch01
1800
1600
1400
1200
1000
800
600
400
200
0
1
2
3
Mean Pixel M04 Ch04
60
45
30
15
0
-15
-30
-45
-60
1
2
3
Intensity MC Ch02
1800000.0
1600000.0
1400000.0
1200000.0
1000000.0
800000.0
600000.0
400000.0
200000.0
0.0
1
2
3
Intensity MC Ch05
525000.00
450000.00
375000.00
300000.00
225000.00
150000.00
75000.00
0.00
-75000.00
1
2
3
Mean Pixel M02 Ch02
600
525
450
375
300
225
150
75
0
1
2
3
Mean Pixel M06 Ch06
300
250
200
150
100
50
0
1
2
3
Intensity MC Ch03
450000.00
375000.00
300000.00
225000.00
150000.00
75000.00
0.00
-75000.00
1
2
3
Intensity MC Ch06
200000.0
150000.0
100000.0
50000.0
0.0
-50000.0
-100000.0
1
2
3
Mean Pixel M03 Ch03
300
250
200
150
100
50
0
1
2
3
Mean Pixel M05 Ch05
600
525
450
375
300
225
150
75
0
1
2
3

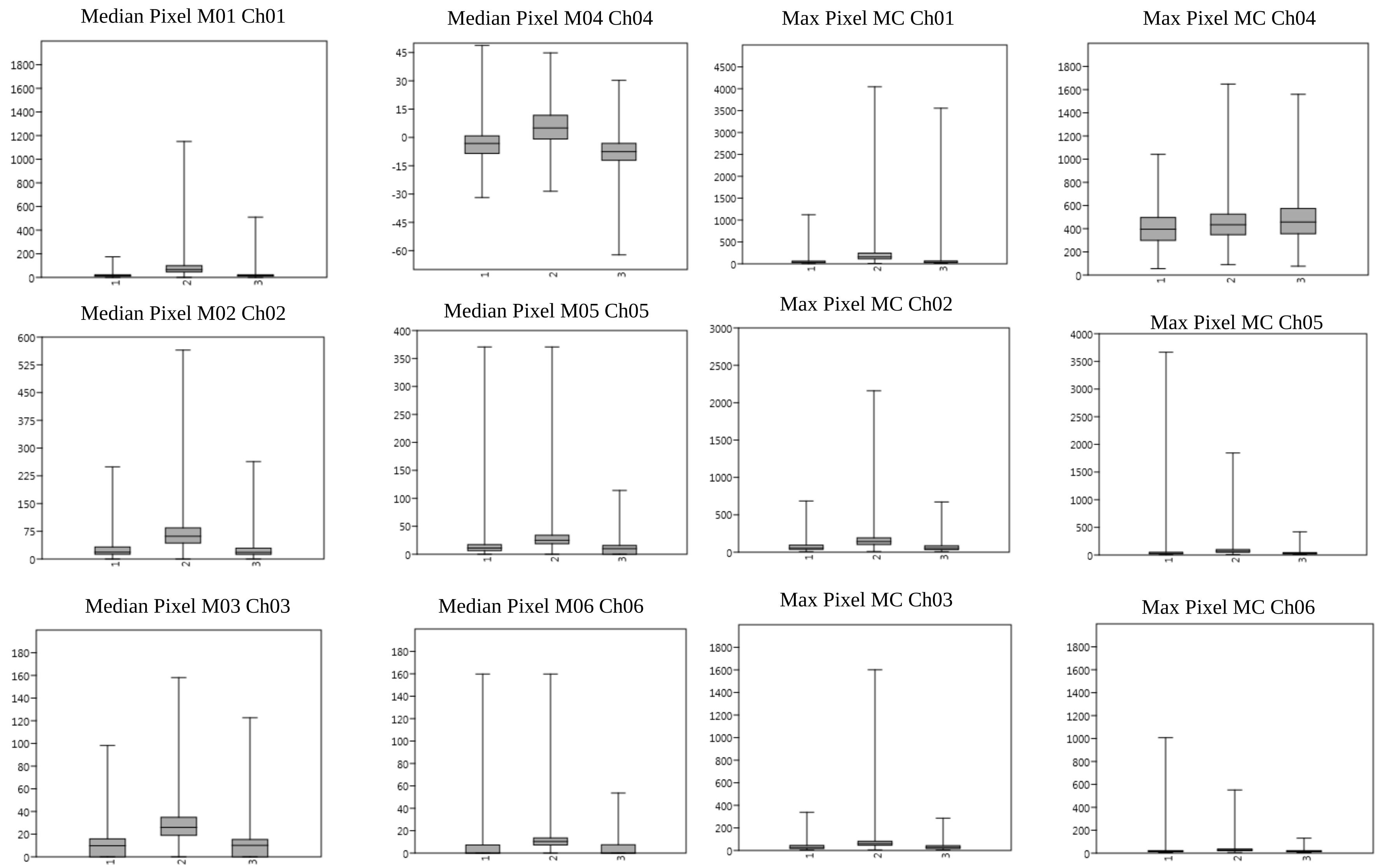

Median Pixel M01 Ch01
0 200 400 600 800 1000 1200 1400 1600 1800
1 2 3
Median Pixel M04 Ch04
-60 -45 -30 -15 0 15 30 45
1 2 3
Max Pixel MC Ch01
0 500 1000 1500 2000 2500 3000 3500 4000 4500
1 2 3
Max Pixel MC Ch04
0 200 400 600 800 1000 1200 1400 1600 1800
1 2 3
Median Pixel M02 Ch02
0 75 150 225 300 375 450 525 600
1 2 3
Median Pixel M05 Ch05
0 50 100 150 200 250 300 350 400
1 2 3
Max Pixel MC Ch02
0 500 1000 1500 2000 2500 3000
1 2 3
Max Pixel MC Ch05
0 500 1000 1500 2000 2500 3000 3500 4000
1 2 3
Median Pixel M03 Ch03
0 20 40 60 80 100 120 140 160 180
1 2 3
Median Pixel M06 Ch06
0 20 40 60 80 100 120 140 160 180
1 2 3
Max Pixel MC Ch03
0 200 400 600 800 1000 1200 1400 1600 1800
1 2 3
Max Pixel MC Ch06
0 200 400 600 800 1000 1200 1400 1600 1800
1 2 3

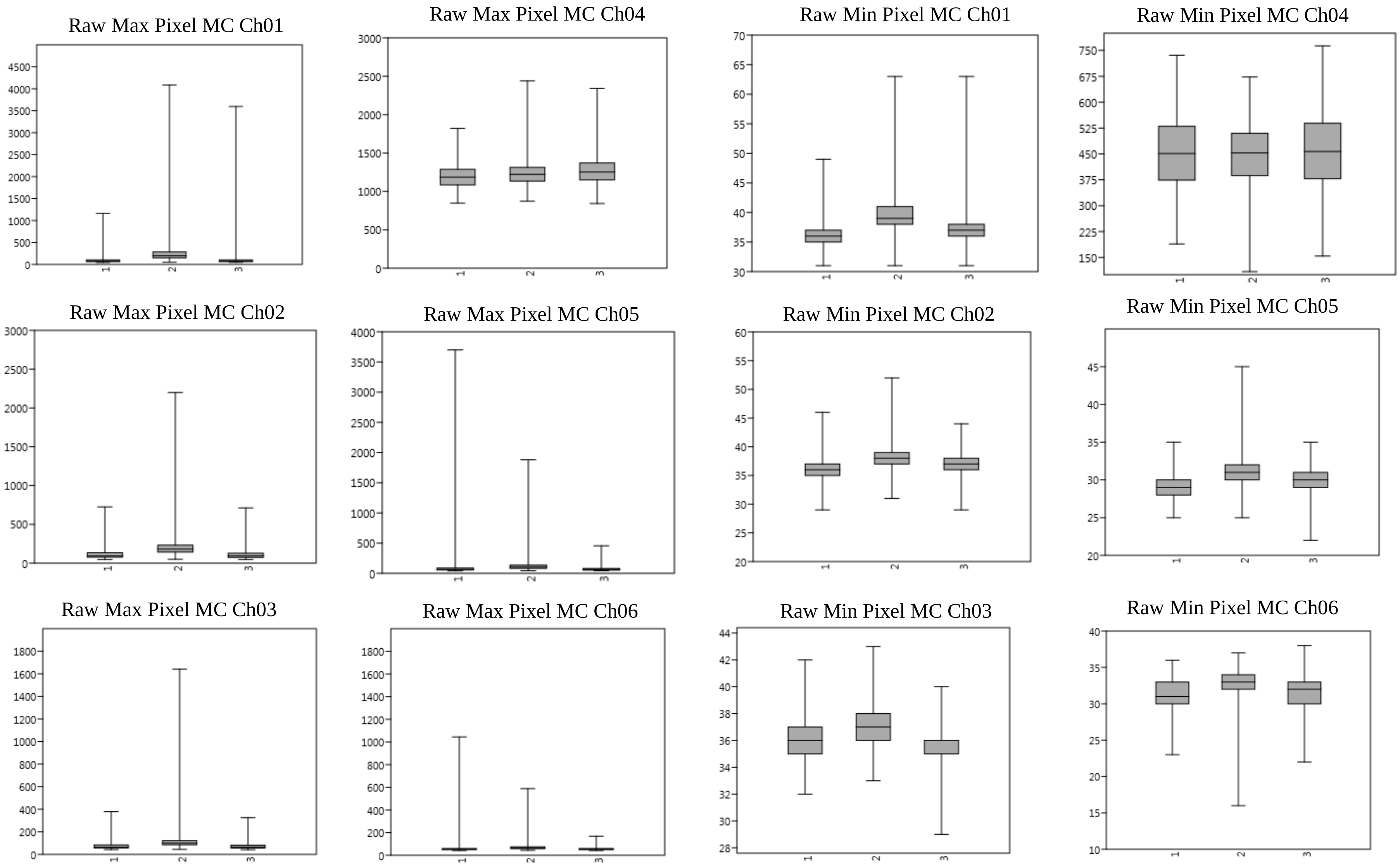

Raw Max Pixel MC Ch01
Raw Max Pixel MC Ch04
Raw Min Pixel MC Ch01
Raw Min Pixel MC Ch04
Raw Max Pixel MC Ch02
Raw Max Pixel MC Ch05
Raw Min Pixel MC Ch02
Raw Min Pixel MC Ch05
Raw Max Pixel MC Ch03
Raw Max Pixel MC Ch06
Raw Min Pixel MC Ch03
Raw Min Pixel MC Ch06

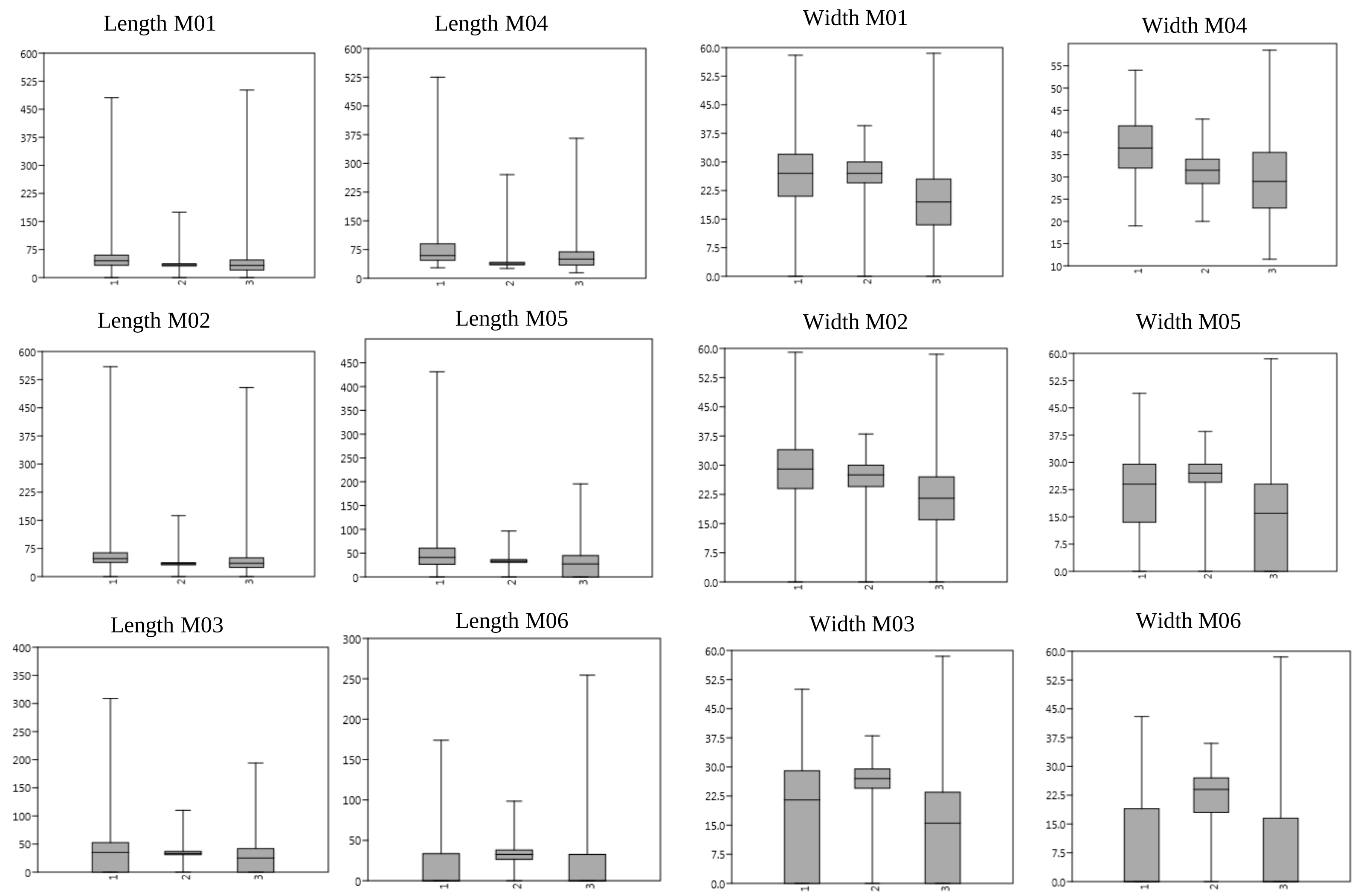

Length M01
Length M04
Width M01
Width M04
Length M02
Length M05
Width M02
Width M05
Length M03
Length M06
Width M03
Width M06

Height M01

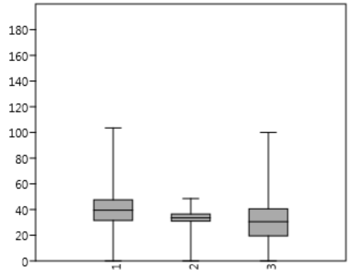

Height M04

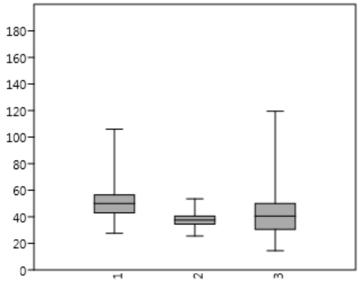

Circularity

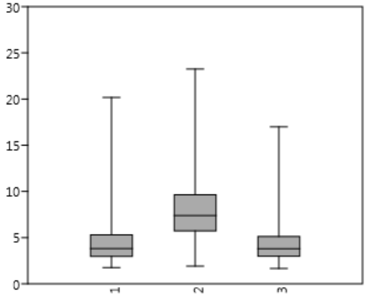

Height M02

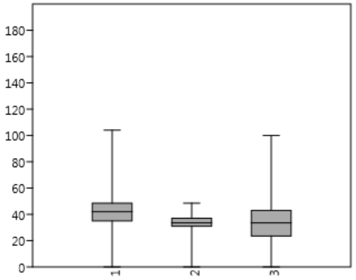

Height M05

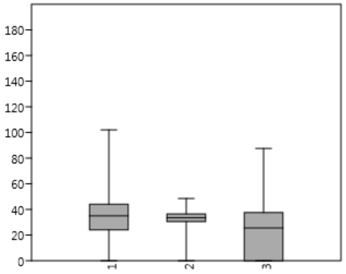

Area MC

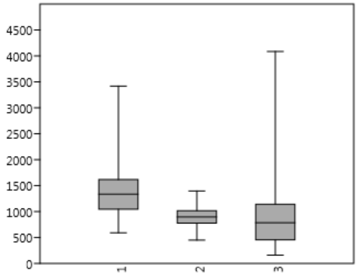

Height M03

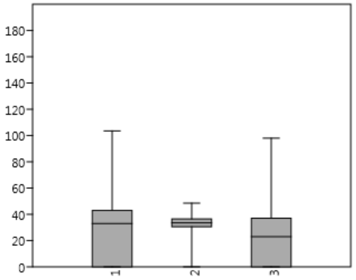

Height M06

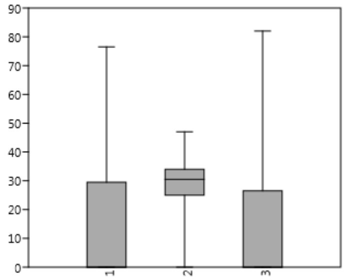

Brightness Detail Intensity R3 M01 Ch01

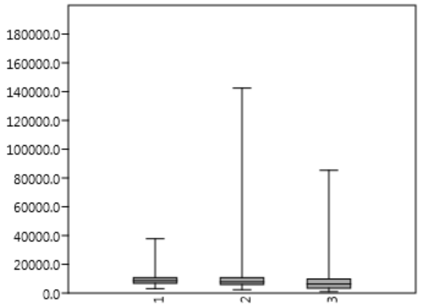

Brightness Detail Intensity R3 M01 Ch02

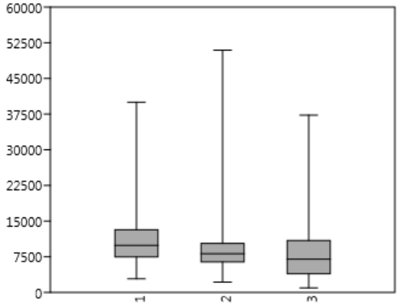

Brightness Detail Intensity R3 M03 Ch03

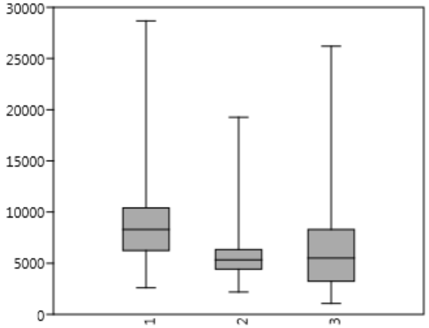

Brightness Detail Intensity R3 M04 Ch04

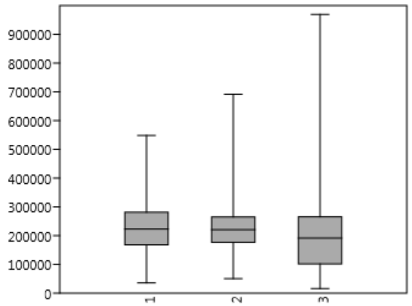

Brightness Detail Intensity R3 M05 Ch05

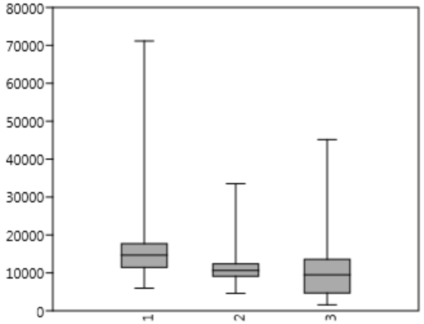

Brightness Detail Intensity R3 M06 Ch06

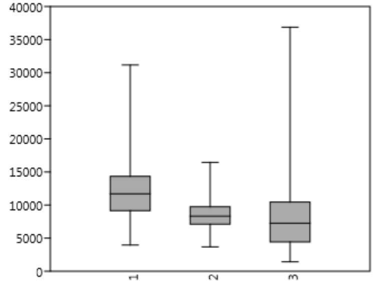

Supplement: S2 Fig — Group 1 = Buccal cells, Group 2 = Epidermal cells, Group 3 = Vaginal cells. (PDF) [file pone.0197701.s002.pdf]
