# Supplementary material for: Rapid differentiation of epithelial cell types in aged biological samples using autofluorescence and morphological signatures
Source: PLoS One. 2018 May 17;13(5):e0197701. doi: 10.1371/journal.pone.0197701 (PMC5957390; doi:10.1371/journal.pone.0197701)
Supplement: S1 Table — (DOCX) [file pone.0197701.s004.docx]

| Buccal | | Contact Epidermal | | Vaginal | |
| --- | --- | --- | --- | --- | --- |
| Sample # | Time Dried | Sample # | Time Dried | Sample # | Time Dried |
| I66 | 24 hrs | L49 | 24 hrs | 1031 | 72 hrs |
| L49 | 24 hrs | N08 | 24 hrs | 2368 | 5 days |
| R47 | 48 hrs | S95 | 24 hrs | 4017 | 6 days |
| 5034 | 48 hrs | I66 | 72 hrs | 1022 | 7 days |
| C58 | 72 hrs | K36 | 4 days | 1028 | 7 days |
| 5001 | 4 days | P22 | 4 days | 4502 | 12 days |
| Y60 | 5 days | R47 | 4 days | 4504 | 12 days |
| Z32 | 5 days | Y60 | 5 days | 5021 | 14 days |
| N08 | 6 days | Z32 | 5 days | 5020 | 16 days |
| B21 | 6 days | Q17 | 5 days | 5005 | 8 weeks |

**S1 Table.** **Tissue type and drying time for each sample.**
