# Supplementary material for: Rapid differentiation of epithelial cell types in aged biological samples using autofluorescence and morphological signatures
Source: PLoS One. 2018 May 17;13(5):e0197701. doi: 10.1371/journal.pone.0197701 (PMC5957390; doi:10.1371/journal.pone.0197701)
Supplement: S2 Table — (PDF) [file pone.0197701.s005.pdf]

Table S2. ANOVA (Tukey HSD) for IFC measurments across cell types

Dependent Variable			Mean Difference (I-J)	Std. Error	Sig.
Area_M01	1	2	-9.70	9.25	0.55
		3	249.74	9.46	0.00
	2	3	259.44	9.11	0.00
Area_M02	1	2	130.04	8.50	0.00
		3	320.14	8.69	0.00
	2	3	190.10	8.37	0.00
Area_M03	1	2	-247.79	9.43	0.00
		3	126.69	9.65	0.00
	2	3	374.48	9.29	0.00
Area_M04	1	2	410.44	9.82	0.00
		3	478.27	10.05	0.00
	2	3	67.83	9.67	0.00
Area_M05	1	2	-170.81	9.04	0.00
		3	189.54	9.25	0.00
	2	3	360.35	8.90	0.00
Area_M06	1	2	-362.55	7.21	0.00
		3	-6.82	7.38	0.63
	2	3	355.73	7.10	0.00
Area_MC	1	2	421.21	9.89	0.00
		3	488.10	10.12	0.00
	2	3	66.88	9.74	0.00
Aspect Ratio_M01	1	2	-0.24	0.01	0.00
		3	-0.01	0.01	0.05
	2	3	0.22	0.01	0.00
Aspect Ratio_M02	1	2	-0.15	0.00	0.00
		3	0.03	0.00	0.00
	2	3	0.18	0.00	0.00
Aspect Ratio_M03	1	2	-0.37	0.01	0.00
		3	0.00	0.01	0.94
	2	3	0.37	0.01	0.00
Aspect Ratio_M04	1	2	-0.13	0.00	0.00
		3	0.00	0.00	0.98
	2	3	0.13	0.00	0.00
Aspect Ratio_M05	1	2	-0.29	0.01	0.00
		3	0.06	0.01	0.00
	2	3	0.35	0.01	0.00

Aspect Ratio_M06	1	2	-0.47	0.01	0.00
		3	-0.02	0.01	0.01
	2	3	0.44	0.01	0.00
Aspect Ratio Intensity_M01_Ch01	1	2	-0.22	0.01	0.00
		3	-0.01	0.01	0.65
	2	3	0.21	0.01	0.00
Aspect Ratio Intensity_M02_Ch02	1	2	-0.14	0.01	0.00
		3	0.03	0.01	0.00
	2	3	0.17	0.01	0.00
Aspect Ratio Intensity_M03_Ch03	1	2	-0.35	0.01	0.00
		3	0.01	0.01	0.57
	2	3	0.35	0.01	0.00
Aspect Ratio Intensity_M04_Ch04	1	2	-0.13	0.00	0.00
		3	0.01	0.00	0.02
	2	3	0.14	0.00	0.00
Aspect Ratio Intensity_M05_Ch05	1	2	-0.27	0.01	0.00
		3	0.06	0.01	0.00
	2	3	0.33	0.01	0.00
Aspect Ratio Intensity_M06_Ch06	1	2	-0.45	0.01	0.00
		3	-0.02	0.01	0.03
	2	3	0.43	0.01	0.00
Contrast_M01_Ch01	1	2	-1.38	0.31	0.00
		3	-2.51	0.31	0.00
	2	3	-1.13	0.30	0.00
Contrast_M02_Ch02	1	2	1.46	0.12	0.00
		3	0.03	0.12	0.98
	2	3	-1.44	0.12	0.00
Contrast_M03_Ch03	1	2	-0.64	0.13	0.00
		3	-0.60	0.13	0.00
	2	3	0.04	0.13	0.95
Contrast_M04_Ch04	1	2	-8.05	0.22	0.00
		3	-7.57	0.23	0.00
	2	3	0.48	0.22	0.07
Contrast_M05_Ch05	1	2	-1.47	0.49	0.01
		3	1.98	0.50	0.00
	2	3	3.45	0.49	0.00
Contrast_M06_Ch06	1	2	-5.02	0.15	0.00
		3	-0.61	0.16	0.00
	2	3	4.41	0.15	0.00

Intensity_MC_Ch01	1	2	-213660.99	5091.02	0.00
		3	14954.98	5209.35	0.01
	2	3	228615.96	5014.95	0.00
Intensity_MC_Ch02	1	2	-96853.14	2494.05	0.00
		3	25735.41	2552.02	0.00
	2	3	122588.55	2456.78	0.00
Intensity_MC_Ch03	1	2	-37869.30	974.95	0.00
		3	10921.89	997.61	0.00
	2	3	48791.19	960.39	0.00
Intensity_MC_Ch04	1	2	-38514.55	988.11	0.00
		3	3074.35	1011.07	0.01
	2	3	41588.90	973.34	0.00
Intensity_MC_Ch05	1	2	-47674.28	1292.31	0.00
		3	10759.07	1322.34	0.00
	2	3	58433.36	1273.00	0.00
Intensity_MC_Ch06	1	2	-16945.64	405.18	0.00
		3	2360.82	414.59	0.00
	2	3	19306.46	399.12	0.00
Mean Pixel_M01_Ch01	1	2	-75.78	1.51	0.00
		3	-3.75	1.55	0.04
	2	3	72.03	1.49	0.00
Mean Pixel_M02_Ch02	1	2	-38.66	0.88	0.00
		3	-0.59	0.90	0.79
	2	3	38.08	0.87	0.00
Mean Pixel_M03_Ch03	1	2	-16.67	0.37	0.00
		3	-0.40	0.38	0.54
	2	3	16.27	0.37	0.00
Mean Pixel_M04_Ch04	1	2	-9.54	0.27	0.00
		3	2.46	0.27	0.00
	2	3	12.00	0.26	0.00
Mean Pixel_M05_Ch05	1	2	-20.06	0.57	0.00
		3	0.27	0.58	0.89
	2	3	20.33	0.56	0.00
Mean Pixel_M06_Ch06	1	2	-8.63	0.21	0.00
		3	-0.88	0.21	0.00
	2	3	7.75	0.21	0.00
Median Pixel_M01_Ch01	1	2	-72.95	1.30	0.00
		3	-2.40	1.33	0.17
	2	3	70.55	1.28	0.00

Median Pixel_M02_Ch02	1	2	-39.37	0.83	0.00
		3	0.25	0.84	0.95
	2	3	39.62	0.81	0.00
Median Pixel_M03_Ch03	1	2	-16.80	0.35	0.00
		3	-0.07	0.36	0.98
	2	3	16.73	0.34	0.00
Median Pixel_M04_Ch04	1	2	-9.19	0.25	0.00
		3	3.73	0.25	0.00
	2	3	12.91	0.24	0.00
Median Pixel_M05_Ch05	1	2	-19.51	0.49	0.00
		3	0.44	0.50	0.65
	2	3	19.96	0.48	0.00
Median Pixel_M06_Ch06	1	2	-8.23	0.19	0.00
		3	-0.72	0.19	0.00
	2	3	7.51	0.18	0.00
Max Pixel_MC_Ch01	1	2	-234.61	6.92	0.00
		3	-15.32	7.08	0.08
	2	3	219.29	6.81	0.00
Max Pixel_MC_Ch02	1	2	-92.16	2.54	0.00
		3	-6.38	2.60	0.04
	2	3	85.79	2.50	0.00
Max Pixel_MC_Ch03	1	2	-34.81	0.98	0.00
		3	-2.86	1.00	0.01
	2	3	31.95	0.96	0.00
Max Pixel_MC_Ch04	1	2	-67.13	4.32	0.00
		3	-75.21	4.42	0.00
	2	3	-8.08	4.25	0.14
Max Pixel_MC_Ch05	1	2	-53.97	1.91	0.00
		3	-0.42	1.96	0.97
	2	3	53.54	1.89	0.00
Max Pixel_MC_Ch06	1	2	-15.61	0.54	0.00
		3	-1.99	0.55	0.00
	2	3	13.62	0.53	0.00
Raw Max Pixel_MC_Ch01	1	2	-235.09	6.92	0.00
		3	-15.82	7.08	0.07
	2	3	219.27	6.81	0.00
Raw Max Pixel_MC_Ch02	1	2	-92.51	2.54	0.00
		3	-6.98	2.60	0.02
	2	3	85.52	2.51	0.00

Raw Max Pixel_MC_Ch03	1	2	-35.03	0.98	0.00
		3	-2.81	1.00	0.01
	2	3	32.22	0.96	0.00
Raw Max Pixel_MC_Ch04	1	2	-66.87	4.37	0.00
		3	-82.78	4.47	0.00
	2	3	-15.91	4.30	0.00
Raw Max Pixel_MC_Ch05	1	2	-54.04	1.92	0.00
		3	-0.53	1.96	0.96
	2	3	53.51	1.89	0.00
Raw Max Pixel_MC_Ch06	1	2	-15.80	0.54	0.00
		3	-2.23	0.55	0.00
	2	3	13.57	0.53	0.00
Raw Min Pixel_MC_Ch01	1	2	-3.63	0.07	0.00
		3	-0.75	0.07	0.00
	2	3	2.88	0.07	0.00
Raw Min Pixel_MC_Ch02	1	2	-2.44	0.05	0.00
		3	-1.05	0.05	0.00
	2	3	1.38	0.05	0.00
Raw Min Pixel_MC_Ch03	1	2	-1.66	0.04	0.00
		3	-0.17	0.04	0.00
	2	3	1.49	0.04	0.00
Raw Min Pixel_MC_Ch04	1	2	22.89	2.80	0.00
		3	-0.89	2.86	0.95
	2	3	-23.78	2.75	0.00
Raw Min Pixel_MC_Ch05	1	2	-2.05	0.05	0.00
		3	-0.64	0.05	0.00
	2	3	1.41	0.05	0.00
Raw Min Pixel_MC_Ch06	1	2	-1.98	0.05	0.00
		3	-0.49	0.05	0.00
	2	3	1.48	0.05	0.00
Length_M01	1	2	14.99	0.75	0.00
		3	14.52	0.77	0.00
	2	3	-0.47	0.74	0.80
Length_M02	1	2	27.61	0.89	0.00
		3	23.07	0.91	0.00
	2	3	-4.54	0.88	0.00
Length_M03	1	2	-0.59	0.66	0.64
		3	8.12	0.67	0.00
	2	3	8.71	0.65	0.00

Length_M04	1	2	44.52	1.19	0.00
		3	26.55	1.22	0.00
	2	3	-17.97	1.17	0.00
Length_M05	1	2	11.69	0.81	0.00
		3	17.84	0.83	0.00
	2	3	6.15	0.80	0.00
Length_M06	1	2	-18.36	0.68	0.00
		3	-2.17	0.70	0.01
	2	3	16.19	0.67	0.00
Width_M01	1	2	-2.90	0.26	0.00
		3	4.92	0.27	0.00
	2	3	7.82	0.26	0.00
Width_M02	1	2	1.20	0.21	0.00
		3	6.69	0.21	0.00
	2	3	5.49	0.20	0.00
Width_M03	1	2	-10.13	0.31	0.00
		3	2.68	0.31	0.00
	2	3	12.81	0.30	0.00
Width_M04	1	2	5.32	0.19	0.00
		3	7.09	0.19	0.00
	2	3	1.77	0.19	0.00
Width_M05	1	2	-6.81	0.29	0.00
		3	5.18	0.29	0.00
	2	3	11.99	0.28	0.00
Width_M06	1	2	-15.09	0.29	0.00
		3	-0.97	0.30	0.00
	2	3	14.12	0.29	0.00
Height_M01	1	2	2.60	0.40	0.00
		3	6.95	0.40	0.00
	2	3	4.35	0.39	0.00
Height_M02	1	2	8.11	0.31	0.00
		3	8.90	0.32	0.00
	2	3	0.78	0.31	0.03
Height_M03	1	2	-7.63	0.47	0.00
		3	4.52	0.48	0.00
	2	3	12.16	0.46	0.00
Height_M04	1	2	12.61	0.30	0.00
		3	9.64	0.30	0.00
	2	3	-2.97	0.29	0.00

Height_M05	1	2	-3.19	0.43	0.00
		3	7.60	0.44	0.00
	2	3	10.79	0.43	0.00
Height_M06	1	2	-17.98	0.43	0.00
		3	-1.84	0.44	0.00
	2	3	16.15	0.43	0.00
Raw Centroid X	1	2	0.34	0.25	0.37
		3	0.15	0.26	0.83
	2	3	-0.19	0.25	0.73
Raw Centroid Y	1	2	5.31	0.41	0.00
		3	1.82	0.43	0.00
	2	3	-3.49	0.42	0.00
Circularity_Object(M04,Ch04,Tight)	1	2	-3.40	0.07	0.00
		3	0.04	0.07	0.85
	2	3	3.45	0.07	0.00
Bright Detail Intensity R3_MC_Ch01	1	2	-2678.25	203.95	0.00
		3	1768.58	208.69	0.00
	2	3	4446.82	200.90	0.00
Bright Detail Intensity R3_MC_Ch02	1	2	1467.11	124.56	0.00
		3	2967.94	127.45	0.00
	2	3	1500.83	122.70	0.00
Bright Detail Intensity R3_MC_Ch03	1	2	2910.28	78.45	0.00
		3	2587.08	80.28	0.00
	2	3	-323.20	77.28	0.00
Bright Detail Intensity R3_MC_Ch04	1	2	-7781.59	2521.47	0.01
		3	32870.53	2580.08	0.00
	2	3	40652.12	2483.80	0.00
Bright Detail Intensity R3_MC_Ch05	1	2	3489.77	120.53	0.00
		3	4926.96	123.33	0.00
	2	3	1437.19	118.73	0.00
Bright Detail Intensity R3_MC_Ch06	1	2	3395.60	95.23	0.00
		3	4325.74	97.45	0.00
	2	3	930.14	93.81	0.00
